# Anti-Cancer Drugs Targeting Protein Kinases Approved by FDA in 2020

**DOI:** 10.3390/cancers13050947

**Published:** 2021-02-24

**Authors:** Jonas Cicenas, Asta Račienė

**Affiliations:** 1 Proteomics Center, Institute of Biochemistry, Vilnius University Life Sciences Center, Sauletekio al. 7, LT-10257 Vilnius, Lithuania; 2 MAP Kinase Resource, Bioinformatics, Melchiorstrasse 9, CH-3027 Bern, Switzerland; 3 Vilnius University Hospital, Santariskiu Klinikos Santariskiu str. 2, LT-08661 Vilnius, Lithuania; astaraciene@yahoo.com

Cancers are a large group of diseases that mostly emerge because of the uncontrollable action of many different genes in human cells. There are possibilities of gene fusions, deletions, amplifications, overexpression, and other abnormalities that lead to the development of cancers. One group of culprits in cancer development are protein kinases, a large family of enzymes catalyzing protein phosphorylation. The human genome contains more than 500 protein kinase genes. Kinases regulate various cellular functions, such as proliferation, cell cycle, apoptosis, differentiation, etc. [1]. Deregulation of kinase activity can result in striking changes in these processes and can be important for the survival and spread of cancer cells [2]. Therefore, many kinases are being investigated as drug targets, for example ABL [3] CDKs [4,5,6], ERBB2 (HER2) [7], AURKs [8,9], MAPKs [10], and many others. Many of those drugs—namely, small-molecule inhibitors or monoclonal antibodies—are already approved by the U.S. Food and Drug Administration (FDA), a federal agency of the Department of Health and Human Services. Many other drugs of this kind are in clinical trials or preclinical development. In this review we will discuss drugs that were approved by the FDA in the 2020. 

Avapritinib (BLU-285) (brand name Ayvakit) (Figure 1a) was approved by the FDA on January 9, 2020, for the treatment of unresectable or metastatic gastrointestinal stromal tumors (GIST). It is used as an inhibitor against human PDGFRA receptor kinase, which has a D842V mutation in this disease. The decision was made relying on results from NAVIGATOR (NCT02508532), a multicenter, single-arm, open-label trial enrolling 43 patients with GIST harboring a PDGFRA exon 18 mutation, including 38 patients with PDGFRA D842V mutations [11].

Selumetinib (AZD6244, ARRY-142886) (brand name Koselugo) (Figure 1a) was approved by the FDA on April 10, 2020, for the treatment of neurofibromatosis type I (NF1), which causes the growth of tumors along nerves in the brain, as well as other parts of the body. It is used as an inhibitor against BRAF kinase with a V600E mutation. The decision was made relying on results from a clinical trial (NCT01362803) of 50 children 2–18 years of age with NF1 [12].

Tucatinib (ONT-380, ARRY-380) (brand name Tukysa) (Figure 1a) was approved by the FDA on April 17, 2020, for the treatment of unresectable or metastatic HER2-positive breast cancer. It is used as an inhibitor of the human ERBB2 receptor kinase. The decision was made relying on results from the HER2CLIMB clinical trial (NCT02614794), a study on tucatinib versus placebo in combination with capecitabine and trastuzumab, enrolling 612 patients [13].

Pemigatinib (INCB054828) (brand name Pemazyre) (Figure 1a) was approved by the FDA on April 17, 2020, for the treatment of advanced/metastatic or surgically unresectable cholangiocarcinoma. It is used as an inhibitor of the human FGFR2 receptor kinase. The decision was made relying on results from the FIGHT-202 (NCT02924376) multicenter, open-label, single-arm trial that enrolled 107 participants with locally advanced or metastatic cholangiocarcinoma with an FGFR2 fusion or rearrangement who had received prior treatment and failed it [14].

Capmatinib (INC280) (brand name Tabrecta) (Figure 1b) was approved by the FDA on May 5, 2020, for the treatment of metastatic non-small cell lung cancer (NSCLC) harboring exon 14 mutations in the MET receptor tyrosine kinase (also known as hepatocyte growth factor receptor). Capmatinib is used as an inhibitor of the human MET receptor kinase. The decision was made relying on results from the GEOMETRY mono-1 trial (NCT02414139), a multicenter, non-randomized, open-label, multicohort study enrolling 334 participants with metastatic NSCLC with confirmed MET exon 14 skipping [15].

Selpercatinib (LOXO-292) (brand name Retevmo) (Figure 1b) was approved by the FDA on May 8, 2020, for the treatment of non-small cell lung cancer, metastatic medullary thyroid cancer or advanced or metastatic thyroid cancer caused by an abnormal RET gene. It is used as an inhibitor of the human RET receptor kinase. The decision was made relying on results from the LIBRETTO-001 clinical trial (NCT03157128) of 702 patients 15–92 years old with certain cancers caused by abnormal RET gene expression [16].

Ripretinib (DCC-2618) (brand name Qinlock) (Figure 1b) was approved by the FDA on May 15, 2020, for the treatment of advanced GIST patients previously treated with imatinib, sunitinib, and regorafenib. It is used as an inhibitor of the human PDGFRA and KIT receptor kinases, usually mutated in GIST. The decision was made relying on results from INVICTUS (NCT03353753), an international, multicenter, randomized, double-blind, placebo-controlled clinical trial that enrolled 129 participants with GIST. [17].

Pralsetinib (BLU-667) (brand name Gavreto) (Figure 1b) was approved by the FDA on September 4, 2020, for the treatment of thyroid cancer, non-small cell lung cancer, and some other tumors. It is used as an inhibitor of the human RET receptor kinase. The decision was made relying on results from the ARROW clinical trial (NCT03037385), which included 220 patients 26–87 years old with NSCLC caused by abnormal RET genes [18].

Margetuximab *(*brand name Margenza) was approved by the FDA on December 16, 2020, for the treatment of HER2-positive breast cancer. It is a chimeric IgG monoclonal antibody medication against HER2 receptor tyrosine kinase. The decision was made based on evidence from a clinical trial (NCT02492711) of 536 patients 27 to 86 years old with HER2-positive metastatic breast cancer who had been previously treated for their metastatic disease [19].

Other notable drugs approved by the FDA since 2014: ramucirumab (brand name Cyramza), a VEGFR2 inhibitor for advanced stomach cancer or gastroesophageal junction carcinoma (2014); palbociclib *(*brand name Ibrance), a CDK4/6 inhibitor for advanced (metastatic) breast cancer (2015); lenvatinib (brand name Lenvima), a VEGFR1/2/3 inhibitor for progressive, differentiated thyroid cancer (2015); cobimetinib (brand name Cotellic), a MEK inhibitor for melanoma in combination with vemurafenib, a BRAF inhibitor (2015); osimertinib (brand name Tagrisso), an EGFR inhibitor for non-small-cell lung carcinomas with specific mutations (2015); necitumumab (brand name Portrazza), an EGFR antibody for advanced (metastatic) squamous non-small cell lung cancer (2015); alectinib (brand name Alecensa), an ALK inhibitor for non-small-cell lung cancer (2015); olaratumab (brand name Lartruvo), a PDGFRA inhibitor for certain types of soft tissue sarcoma (2016); ribociclib (brand name Kisqali), a CDK4/6 inhibitor for advanced breast cancer (2017); brigatinib (brand name Alunbrig), an ALK and EGFR inhibitor for non-small-cell lung cancer (2017); copanlisib (brand name Aliqopa), a PI3K inhibitor for relapsed follicular lymphoma (2017); abemaciclib (brand names Verzenio, Verzenios, and Ramiven), CDK4/6 inhibitors for advanced or metastatic breast cancers (2017); acalabrutinib (brand name Calquence), a BTK inhibitor for mantle cell lymphoma (2017); binimetinib (brand name Mektovi), a MEK inhibitor for unresectable or metastatic melanoma (2018); encorafenib (brand name Braftovi), a MEK inhibitor for unresectable or metastatic melanoma (2018); duvelisib (brand name Copiktra), a PI3K inhibitor for refractory chronic lymphocytic leukemia, small lymphocytic lymphoma, and follicular lymphoma (2018); dacomitinib (brand name Vizimpro), an EGFR inhibitor for metastatic non-small-cell lung cancer (2018); lorlatinib (brand names Lorbrena and Lorviqua), an ALK and ROS1 inhibitor for metastatic non-small cell lung cancer (2018); gilteritinib (brand name Xospata), an AXL inhibitor for relapsed or refractory acute myeloid leukemia (2018); erdafitinib (brand name Balversa), an FGFR inhibitor for locally advanced or metastatic bladder cancer (2019); alpelisib (brand name Piqray), a PI3K inhibitor for breast cancer (2019); pexidartinib (brand name Turalio), an inhibitor of CSF1, KIT, and FLT3 for symptomatic tenosynovial giant cell tumor (2019); entrectinib (brand name Rozlytrek), an inhibitor of ALK, ROS1, TKI, and TRKA/B/C for metastatic non-small cell lung cancer (2019); and zanubrutinib (brand name Brukinsa), an inhibitor of BTK for mantle cell lymphoma (2019).

Current progress in the field of small-molecule inhibitors of kinases has led to the development of a number of compounds, with various choices for each target molecule, as well as antitumor potencies and specificity for different types/stages of cancers. On the other hand, there are already a lot of antibodies targeting kinases available. Nevertheless, there is still ample room for additional development. Hopefully, there will be a lot of new anti-kinase drugs that can be used either as monotherapy or combined with other anti-cancer medications in the future.

## Figures and Tables

**Figure 1:**
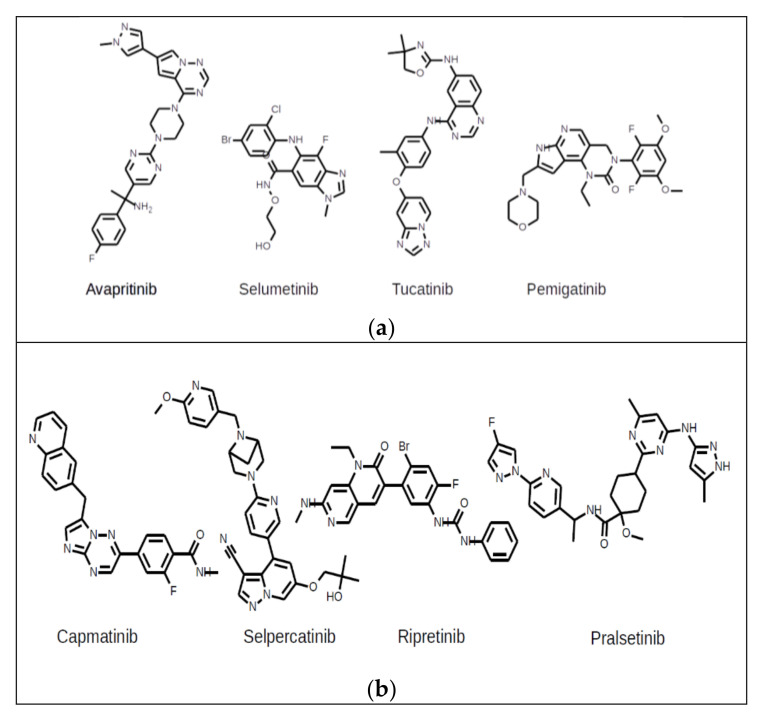
(**a**) Avapritinib, Selumenitib, Tucatinib, Pemigatinib. (**b**) Capmanitinib, Selpercatinib, Ripretinib, Prasetinib.
